# 4084 Predictors of mortality among non-severe hemophilia A patients in the United States

**DOI:** 10.1017/cts.2020.139

**Published:** 2020-07-29

**Authors:** Ming Y Lim, Dunlei Cheng, Nigel Key

**Affiliations:** 1 University of Utah School of Medicine; 2 ATHN; 3 University of North Carolina

## Abstract

OBJECTIVES/GOALS: To determine predictors of mortality in non-severe hemophilia A (NSHA) patients. METHODS/STUDY POPULATION: The ATHNdataset was used to identify NSHA patients who have authorized the sharing of their demographic and clinical information for research. Factors examined included race, ethnicity, hemophilia severity, Hepatitis B, Hepatitis C and HIV infections. A mortality rate was calculated for each factors examined. The relative risk of death between patients in different categories of the factors was assessed by using the ratio of these mortality rates. To adjust for the effects of all of the studied factors with mortality, a multivariate analysis was performed using logistic regression. All hypothesis testing was two-tailed, with a significance level of .05. RESULTS/ANTICIPATED RESULTS: A total of 6,606 NSHA patients were followed for an average of 8.5 years. During 56,064 person years of observation, 136 (2.1%) NSHA patients died; 20% of deaths were malignancy-related. Mortality rates were similar across racial group. Hispanic patients were 60% less likely to die than non-Hispanic patients (p = 0.006). Patients with Hepatitis C infection and HIV infection were 7 times as likely to die compared to those without infections (p<0.001). After adjusting for the effects of all examined factors in a multivariate analysis, patients with hepatitis C and HIV infection remain significantly associated with increased mortality at 6.1 times and 3.6 times the risk, respectively. DISCUSSION/SIGNIFICANCE OF IMPACT: Despite significant improvement in the therapeutic approaches for infectious diseases, Hepatitis C and HIV infections remain strong predictors of mortality in this NSHA cohort. CONFLICT OF INTEREST DESCRIPTION: N/A.

